# Crosstalk among hormones in barley spike contributes to the yield

**DOI:** 10.1007/s00299-019-02430-0

**Published:** 2019-05-28

**Authors:** Helmy M. Youssef, Mats Hansson

**Affiliations:** 1 Faculty of Agriculture, Cairo University, Giza, 12613 Egypt; 2 Department of Biology, Lund University, Sölvegatan 35B, 22362 Lund, Sweden

**Keywords:** Barley spike, Barley yield, Hormones crosstalk, IAA, GA, ABA, CK

## Abstract

**Key message:**

The hormonal ratios along the barley spike regulate the development, atrophy and abortion of the spikelets and could be the mechanism by which the barley spike adapts its yield potential.

**Abstract:**

Barley (*Hordeum vulgare* L.) is one of the oldest cereal crops known to be cultivated since about 10,000 years. The inflorescence of cultivated barley is an indeterminate spike that produces three single-flowered spikelets at each rachis node which make it unique among the grasses. The yield production in barley is predominantly controlled by very important parameters such as number of tillers and number of spikelets per spike. These two parameters are negatively correlated. Therefore, studying the biological and genetics of the spikelet development during the spike developmental stages is essential for breeding programs. Here we summarize our current understanding of the crosstalk between hormones such as auxin, cytokinin, gibberellin and abscisic acid along the spike and what is their role in regulating spike and spikelet development in barley. We conclude that the hormonal ratios at the apical, central, and basal sections of the spike not only regulate the spike developmental stages, but also the development, atrophy, and abortion of the spikelets. This hormonal dependent modification of the grain number along the spike could be the mechanism by which the barley spike adapts its yield potential.

Two of the most important cereal crops, bread wheat (*Triticum aestivum* L.) and barley (*Hordeum vulgare* L.), produce their grains in a very similar inflorescence called spike (Kirby and Appleyard 1987). The spike is formed from the inflorescence meristem through cell divisions to produce a main stem (rachis) and a spikelet meristem at each rachis node. The important morphological differences between wheat and barley are at the spikelet level. In wheat, the spike is determinate (has a terminal spikelet at the apex) while the single spikelet at each rachis node is indeterminate and produces multiple florets. Conversely, the barley inflorescence is indeterminate, but at each rachis node there is a triplet of spikelets (one central and two lateral), which are determinate and contains only one floret each. The barley spike reaches its final size at the anthesis stage, whereby florets within the sessile spikelets attached to the rachis produce grains. While the wild progenitor of cultivated barley is characterized as two-rowed barley with a central fertile spikelet and two sterile lateral spikelets, domestication and breeding produced two types of cultivated barley; the two-rowed type and a six-rowed type where the central as well as the two lateral spikelets are fertile and set seeds (Sakuma et al. 2011).

The number of seeds per spike is a very important parameter for the yield of cereal crop plants. Therefore, improved seed yield is a key objective of many cereals breeding programs (Alqudah and Schnurbusch 2013). The maximum yield potential per spike is represented by the number of spikelets per spike at the awn primordium stage (Kirby and Appleyard 1987). After this stage, spikelets are reduced and start to abort at both ends of the spike. At the awn primordium stage six-rowed barley displays more floret primordia per spike than two-rowed barley (Kirby and Appleyard 1987; Arisnabarreta and Miralles 2006). The growth and development of the inflorescence, as well as the duration of the different developmental phases, are influenced by phytohormones such as auxin (IAA), cytokinin (CK), gibberellin (GA) and abscisic acid (ABA) (Su et al. 2011; Matsoukas 2014; Pearce et al. 2013; Youssef et al. 2017).

The role of hormones in regulating floral organ patterning and phase duration during barley inflorescence and shoot development was described recently in the previous study (Youssef et al. 2017). The gradient of spikelets at different developmental stages along the spike inspired the analysis of concentrations of IAA, CK, GA and ABA in different sections of the spike (Fig. 1). The apical, central and basal sections of immature spikes at the green anther stage contain different concentrations of these hormones. While CK was found to accumulate in the tip of the spike, highest concentrations off IAA were measured in the basal sections. This is in line with what is known about the involvement of CK in organ development from meristematic tissues, where high concentrations of CK regulates the expression of genes encoding IAA influx (like *AUXIN RESISTANT 2; LAX2*) and efflux carriers (*PINs*), causing low concentrations of IAA in the apical part through the relocation of IAA (Dello Ioio et al. 2008; Ruzicka et al. 2009; Zhang et al. 2013). In contrast, biosynthesis of GA, as well as responses to GA, such as germination and flowering are known to be not affected by CK (Greenboim-Wainberg et al. 2005). The low accumulation of GA in the apical part of the spike could be due to the low accumulation of IAA (Dorcey et al. 2009). In experiments with pea (*Pisum sativum* L.), the apical meristem was removed to reduce the IAA content in the rest of the plant (Ross and O’Neill, 2001). The decapitated pea plant had reduced transcript levels of *PsGA3ox1* and consequently reduced levels of GA_1_, the active form of GA. In contrast, the decapitation increased the transcript level of *PsGA2ox1*, which encodes the enzyme converting active GA_1_ and GA_20_ to in-active GA_29_ and GA_8_, respectively (Ross et al. 2000). The same authors proposed that IAA positively affected GA biosynthesis. This might also explain the finding of low concentrations of GA in the apical part of the spike because of the low concentration of IAA (Fig. 1). GA has an antagonistic relation to ABA (Gomez-Cadenas et al. 2001). It was found high concentration of ABA in the apical part of the spike where there are low amounts of GA.

**Fig. 1:**
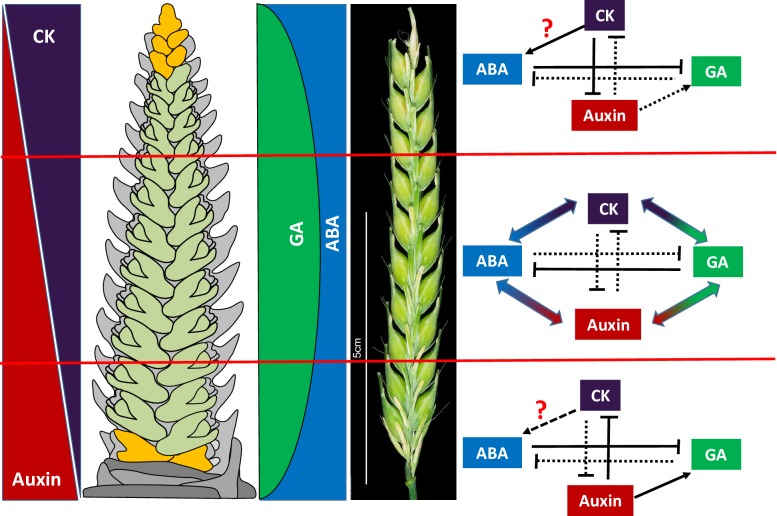
Hormonal crosstalk and their relations along the barley spike sections; apical, central, and basal and their effect on spikelet development. Black solid line with arrowhead indicates the activation/promoting of the hormone biosynthesis. Black solid line with straight end indicates suppression of hormone biosynthesis. Black dots line indicates loss of promoting/suppression effect on hormones biosynthesis. Double-head-arrow indicates balance in the effect among the hormones. The barley spike photo is from barley Bowman cultivar. Cytokinin (CK), gibberellin (GA) and abscisic acid (ABA)

In contrast to the apical part, IAA has a major role in hormonal crosstalk at the basal section of the spike. The high concentration of IAA is indirectly responsible for the low concentration of CK in this part of the spike. IAA regulates the strigolactone biosynthesis gene *More Axillary Growth4* (*MAX4*) (Sorefan et al. 2003). Strigolactones, in turn, negatively regulate CK biosynthesis through the *Isopentenyltransferase1* (*IPT1*) gene (Cai et al. 2018). Additionally, IAA is directly involves in down-regulating the expression of *IPT*, which is a CK biosynthetic gene (Ferguson and Beveridge, 2009). This kind of crosstalk between IAA and CK, either in the apical or basal sections, might maintain the IAA and CK gradients in an inverse basal–apical manner through the spike sections to properly pattern the spike (Youssef et al. 2017; Zwirek et al. 2018). Again, the antagonistic relation between GA and ABA appears an apt explanation for the high and low concentrations of ABA and GA, respectively, in the basal part of the spike.

In the central part of the spike, it was found more balanced concentrations of IAA and CK. At the same time, it was clear that GA is more abundant than ABA and could activate ABA catabolism causing the ABA reduction (Liao et al. 2018). Boden et al. (2014) noted that GA accelerates the spikelet initiation, promotes flowering, and is essential for normal flowering of spring barley under inductive photoperiods. Higher concentrations of GA and lower concentrations of ABA allow the development of fertile florets and grain setting in the central part of the spike (Wang et al. 1999; Cao et al. 2000; Boden et al. 2014). At the green anther stage, relatively late in the development of the spike, the apical and basal spikelet primordia are subjected to atrophy and degradation through the influence of high concentrations of ABA in these regions of the spike (Wang et al. 2001). Studying the transcriptional responses in *six*-*rowed spike3* (*vrs3.f*) barley mutant, Bull et al. (2017) found that the balance of plant hormones plays an important role in regulating lateral spikelet fertility and subsequently affects the barley plant yield. Thus, we conclude that not a single hormone, but the hormonal ratios at the apical, central, and basal sections of the spike are regulating the spike developmental stages and the development, atrophy, and abortion of the spikelets (Fig. 1). This hormonal dependent modification of the grain number along the spike could be the mechanism by which the barley spike adapts its yield potential. The resemblance between barley and wheat spikes suggested that wheat spike might has the same hormonal crosstalk and function along the spike, but this need to be confirmed by more research experiments.

## Author contribution statement

HMY conceived the idea of the manuscript; HMY and MH wrote the manuscript.
